# The Extracellular MicroRNAs on Inflammation: A Literature Review of Rodent Studies

**DOI:** 10.3390/biomedicines10071601

**Published:** 2022-07-05

**Authors:** Seri Lee, Jade Heejae Ko, Seung-Nam Kim

**Affiliations:** 1 College of Korean Medicine, Dongguk University, Goyang 10326, Korea; serilee99@gmail.com (S.L.); jadeko@dongguk.edu (J.H.K.); 2 Graduate School, Dongguk University, Seoul 04620, Korea

**Keywords:** extracellular vesicle, inflammation, organ injury, immune dysfunction, metabolic syndrome, neurological disease, arthritis, cancer

## Abstract

Inflammation is an indispensable biological process stimulated by infection and injuries. Inflammatory mechanisms related to extracellular vesicles (EVs), which are small membrane structures carrying various molecules, were summarized in this review. Emerging evidence from animal studies has highlighted the role of EVs in modulating inflammatory responses, by transporting various molecules involved in host defense. In this review, we have discussed the role of EV miRNAs in inflammation. Rodent studies associated with extracellular miRNAs in inflammatory diseases, published from 2012 to 2022, were explored from PUBMED, EMBASE, and MEDLINE. A total of 95 studies were reviewed. In summary, EV-associated miRNAs play a key role in various diseases, including organ injury, immune dysfunction, neurological disease, metabolic syndrome, vesicular disease, arthritis, cancer, and other inflammatory diseases. Diverse EV-associated miRNAs regulate inflammasome activation and pro- and anti-inflammatory cytokine levels by targeting genes.

## 1. Introduction

The inflammatory response is a rapid and complex physiological process that involves defense mechanisms acting against infections and injuries [1,2]. Inflammation is often regarded as a failure of homeostasis between the host and immune cells. Dysregulation of the inflammatory response underlie various pathological conditions, including chronic inflammation [3], autoimmunity [4], neurodegenerative diseases [5], and cancer [6]. Previous discoveries in inflammatory processes have highlighted the physiological and cellular basis of inflammation under experimental conditions using bacterial lipopolysaccharide (LPS), peptidoglycan, and viral double-stranded RNA [7]. Intracellular signals sent to immune cell nuclei are followed by initial inflammatory cues, which stimulate various transcriptional changes [8]. In addition to the discovery of molecular mechanisms of regulation and initiation of inflammatory responses, a new perspective on inflammatory processes was revealed in recent decades, with the emerging interest in the discovery of mammalian microRNAs (miRNAs) and extracellular vesicles (EVs) [9,10].

EVs are small vesicles (30–10,000 nm in diameter) that can be categorized into the following three types according to their size and biogenesis: exosomes, microvesicles, and apoptotic bodies [11]. EVs are evident in almost all living cells and have been gaining attention as a novel mediator of communication between cells and numerous biological processes and regenerative properties [12,13]. Evs carry various molecules, such as proteins, mRNA [14], long non-coding RNA, circular RNAs, RNA, and miRNAs [10]. miRNAs are endogenous non-coding RNA molecules that use exosomes as carriers to achieve intercellular communication and regulation of protein biosynthesis, while being protected from degradation in the harsh extracellular environment [15]. Extracellular miRNAs have numerous functions in inflammatory processes [16,17], cell migration [18], apoptosis [19], and proliferation [20]. From the perspective of inflammatory responses, various extracellular miRNAs have recently been reported to be expressed in immune cells, affecting the magnitude of their responses [21]. Moreover, the structural stability of extracellular miRNAs has been widely recognized; extracellular miRNAs are now considered potential noninvasive biomarkers for inflammatory disease monitoring and prognosis [22].

This report presents an overview of the recent studies on extracellular miRNAs, with a focus on their role in inflammatory diseases in animals.

## 2. Study Methods

### 2.1. Literature Search

All relevant studies were initially searched on EMBASE, MEDLINE and PUBMED database using the following search keywords: “extracellular vesicles”, “exosome”, “inflammatory diseases”, and “microRNA”. We included rodent studies published from November 2012 to April 2022 and overlapping studies were excluded. Eventually, we identified 320 potentially relevant literatures for further eligibility assessments.

### 2.2. Study Selection

**Figure 1:**
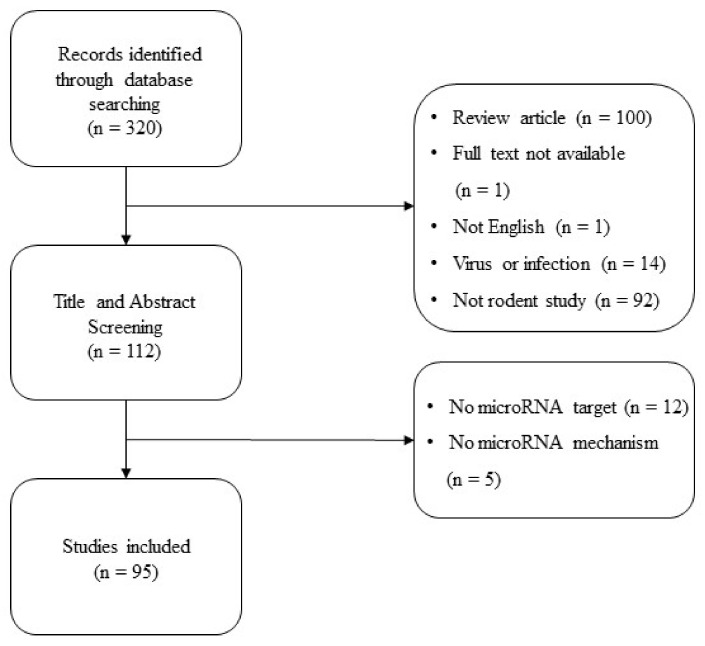
Flowchart of study selection.

Three authors (S.L., J.H.K. and S.-N.K.) independently assessed the 320 literatures and a total of 208 studies were excluded based on the following exclusion criteria: (1) review article (*n* = 100); (2) full text not available (*n* = 1); (3) not English (*n* = 1), (4) virus or infection-induced experiment model (*n* = 14), (5) not a rodent study (*n* = 92). Title and abstract screening were performed and six studies with no specific microRNA target (*n* = 12) and five studies with no mention of microRNA mechanism were excluded. As shown in Figure 1, this study ultimately included a total of 95 articles for further analyses.

## 3. Main Text

### 3.1. Organ Injuries

Among the 95 articles, 35 articles were related to organ-injury-associated miRNAs in exosomes. The uncontrolled inflammatory response is one of the major factors among various causes of organ injuries. The miRNAs are known to target mRNAs and modulate the level of protein expression encoded by these mRNAs. We attempted to determine how miRNAs in exosomes influence organ injury-related diseases, including myocardial infarction, liver failure, ulcerative colitis, and acute lung injury (Table 1).

#### 3.1.1. Liver Injury

In alcoholic and inflammatory liver diseases, serum/plasma miR-122 and miR-155 increased during drug-induced liver injury, and these miRNAs were present in the protein-rich fraction [23]. In a mouse model with autoimmune hepatitis (AIH) induced by hepatic injection of S100 protein, miR-223 in the exosomes derived from bone marrow mesenchymal stem cells (BMSCs) protected the liver from injury and inhibited NLRP3 activation that causes hepatic damage and liver dysfunction [24]. In hepatic fibrogenesis or fibrosis in a carbon tetrachloride-or thioacetic acid-induced liver injury mouse model, EVs of normal mice suppressed hepatocyte death and circulating pro-inflammatory cytokine levels. miR-34c, -151, -483, -532, and -687, which are highly expressed in EVs, have therapeutic effects in injured hepatocytes [25]. In acute liver failure induced in mice by LPS and D-GalN, miR-17 in AMSC-derived exosomes suppressed NLRP3 inflammasome activation by targeting TXNIP [26]. In a high-fat high-cholesterol diet-fed-induced NAFLD rat model, more exosomes were released, contained more miR-192, and the levels of M1-specific cytokines, such as iNOS, IL-6, and TNF-α, increased. miR-192 in exosomes from hepatocytes activated pro-inflammatory macrophages via Rictor/Akt/FoxO1 signaling [27]. In another experimental AIH mouse model, miR-223 carried in MSC exosomes attenuated liver injury and inflammatory responses [28]. In an endotoxemia and chemical liver injury induced by LPS, miR-455exosomes from hUC-MSCs attenuated macrophage infiltration and cured liver damage via PI3K signaling [29].

#### 3.1.2. Lung Injury

In an acute lung injury (ALI) mouse model established by cecal ligation puncture (CLP), miR-125 in exosomes derived from endothelial cells promoted VEGF expression, inflammatory response, improved pathological changes, restrained lung water content, protein content in bronchoalveolar lavage fluid, and cell apoptosis by targeting TOP2A [30]. In an LPS-induced ALI rat model, miR-384 in exosomes derived from BMSC alleviated pathological changes in lung, pulmonary vascular permeability, and attenuated the inflammatory response by targeting Beclin-1 [31]. In a sepsis-induced lung injury mouse model and an LPS-induced AEC damage model, ADSCs exosomes promoted autophagy activation through the delivery of circ-Fryl and the regulation of the miR-490/SIRT3 pathway [32]. In a mouse model of CLP-induced septic lung injury, miR-16 in exosomes developed from ADSCs relieved lung injury and promoted macrophage polarization by suppressing TLR4 [33]. In another study on an LPS-induced ALI mouse model, miR-377 in exosomes from hucMSCs suppressed bronchoalveolar lavage, inflammatory factors, and ameliorated lung injury by targeting RPTOR [34]. In a hyperoxia-induced ALI model established using LPS or *K. pneumoniae*, microvesicles containing miR-223/142 targeted lung macrophages and suppressed inflammatory lung responses by blocking N1rp3 and Asc [35]. In another LPS-induced ALI rat model, miR-22 in exosomes derived from UCB-MSCs suppressed pathological changes, apoptosis, NF-κB expression, and oxidative stress response by reducing FZD6 levels [36].

#### 3.1.3. Heart Injury

miR-133-MSC transplantation improved cardiac function, and miR-133-overexpressing MSCs repressed cardiac expression of *snail-1* and reduced inflammation and fibrosis in the infarcted heart in a rat myocardial infarction model [37]. βARKct EVs altered pro- and anti-inflammatory cytokine levels and prevented heart failure in a myocardial infarction or catecholamine toxicity mouse model. The miRNA profiling revealed that miR-7004 and mi7-7b were upregulated in βARKct present in EVs [38]. In a rat model of hypoxia-induced H9c2 myocardial cell injury, miR-126-enhanced ADSC-derived exosomes decreased the myocardial injury area of infarction, cardiac fibrosis, and inflammatory cytokine expression [39]. In a doxorubicin/trastuzumab-induced cardiac toxicity rat model, cardiac progenitor cell-derived exosomes were highly enriched in miR-146a that prevented myocardial fibrosis, CD68+ inflammatory cell infiltration, inducible nitric oxide synthase expression, and left ventricular dysfunction [40]. In an acute myocardial infarction rat model produced by surgical ligation of the left anterior descending coronary artery, exosomes from miR-146a-ADSCs promoted myocardial cell apoptosis, inflammatory response, and fibrosis, and attenuated myocardial infarction by downregulating EGR1 [41]. Using the same rat model, cardioprotection was observed by exosomes from miR-25-MSCs, and exosomes attenuated myocardial infarction by targeting EZH2 and pro-apoptotic proteins [42]. In a sepsis-induced mouse with myocardial infarction, miR-24 in exosomes derived from M2 macrophages had cardioprotective effects and alleviated myocardial injury by suppressing Tnfsf10 [43]. miR-223 targeted SEMA3A and STAT3, and this miRNA in mice exosomes, had cardioprotective effects in CLP-induced sepsis [44]. In a mouse model of myocardial infarction caused by coronary artery ligation, exosomes overexpressing miR-129 showed enhanced cardiac function and production of inflammatory cytokines, and inhibited apoptosis and fibrosis by targeting HMGB1 [45]. In angiotensin II-induced hypertrophy in rat cardiomyocytes, hypertrophic cardiomyocyte-derived exosomes regulated macrophage activation and induced phosphorylation of ERK, JNK, and p38 through the miR-155-mediated MAPK pathway [46].

#### 3.1.4. Bowel Disease

In a dextran sulfate sodium (DSS)-induced colitis mouse model, miR-378 carried by hucMSC exosomes attenuated colitis by regulating macrophage pyroptosis and inhibiting NLRP3 inflammasome activation [47]. Moreover, miR-590 carried by M2 macrophage exosomes suppressed inflammatory signals and promoted epithelial repair via the LATS1/YAP/β-catenin signaling axis [48]. Another study on the same model showed that miR-21a in M1 exosomes attenuated DSS-induced enteritis by decreasing the expression of E-cadherin and subsequent activation of ILC2s via KLRG1/GATA-3 [49]. In a rat small bowel transplantation model of allograft rejection, miR-200b in exosomes derived from heme oxygen-1 (HO-1)-modified BMSCs alleviated inflammatory injury of intestinal epithelial cells (IECs) by targeting Hmgb3 [50].

#### 3.1.5. Kidney Injury

In the ischemia/reperfusion-injured mouse kidney, miR-23a-enriched exosomes from hypoxic tubular epithelial cells promoted tubulointerstitial inflammation in mice by inhibiting A20; hence, miR-23a inhibition suppressed renal tubulointerstitial inflammation [51]. In a lethal renal ischemia/reperfusion injury rat model, miR-146a, in exosomes derived from USC, inhibited injury via IRAKI and inhibited the activation of NF-κB signaling [52]. A mouse model that was administered a high-fat diet and streptozotocin injection showed that inhibiting Rab27a attenuated inflammation through the miR-26a/CHAC1/NF-κB pathway in renal proximal tubular epithelial cells [53]. In a kidney injury mouse model induced by CLP, miR-21 expression in exosomes extracted from the serum of mice with limb remote ischemic preconditioning in remote organs attenuated sepsis-induced renal injury and regulated the PDCD4/NF-κB and PTEN/AKT pathways [54].

#### 3.1.6. Other Organs

In a taurocholate-induced acute pancreatitis rat model, pro-inflammatory miR-155 was increased in plasma exosomes, and miR-122 and miR-21 were decreased compared to that in plasma control exosomes. The levels of miRNAs in pancreatitis-associated ascitic fluid exosomes were similar to those in plasma control exosomes. Plasma exosomes had higher pro-inflammatory activity in macrophages [55]. In a rat model of urethral stricture generated with TGFβ1 injection, miR-146a in exosomes derived from TNF-α-treated MSCs inhibited fibroblast activation and suppressed the inflammatory response, TRAF6, IRAK1, and NF-κB signaling [56]. In an acute uterine injury mouse model induced by LPS, miR-223 enriched BMSC-Exos degraded NLRP3 via interaction with endothelial progenitor cells and suppressed LPS-induced cell pyroptosis [57].

### 3.2. Immune Dysfunction

Ten articles that we explored were related to the immune dysfunction of miRNAs in exosomes. Immune dysfunction is a disorder of the immune system that includes sepsis and asthma. Recent studies have investigated the roles of miRNAs in immune dysfunction and the associated diseases. We have now organized each miRNA that was introduced as the target of different immune dysfunction studies (Table 2).

For a Rab27KO mouse model that displays a chronic, low-grade inflammatory condition, exosomes carrying miR-155 can rescue LPS responsiveness, and the reduction in miR-155 targeting SHIP1 and IRAK-M is involved in this rescue [58]. In an intestinal lavage of a septic mouse model, pro-inflammatory cytokines TNF-α and IL-17A were suppressed by septic-EV injection, and pro-inflammatory cytokines were targeted by multiple miRNAs upregulated by sepsis-induced exosomes. IEC-derived luminal EVs carry miRNAs that can alleviate pro-inflammatory responses [59]. In a CLP mouse model with severe sepsis, miR-146a, miR-9, and miR-155 functioned as pro-inflammatory messengers. Choroid plexus-derived EVs into CSF transferred pro-inflammatory messages to recipient brain cells, and blockage of EV secretion inhibited brain inflammation [60]. Using mouse chimeras with Rab27KO EV-deficient T cells, miR-20a, miR-25, and miR-155, which are carried in T-cell EV-modulated key mRNA in B cells, promote proliferation, survival, and transfer of EV-miRNA-controlled germinal center reaction and antibody production [61]. In a CLP rat model of sepsis, miR-1 increased in exosomes, and it inhibited proliferation, and promoted apoptosis and cytoskeleton contraction via SERP1 [62]. A mouse model of asthma induced by ovalbumin and miR-370 carried by M2 macrophage-derived exosomes alleviated asthma progression by inhibiting the FGF1 and MAPK/STAT1 signaling pathways [63]. In a mouse model of colitis and systemic inflammation, exosomes transferring let-7d from Treg cells to Th1 cells contributed to the inhibition and suppression of systemic disease [64]. In another ovalbumin-induced asthma mouse model, miR-188 in exosomes derived from hBM-MSCs suppressed the proliferation of BSMCs and lung injury through the JARID2/Wnt/β-catenin axis [65]. In CLP-induced sepsis, miR-146a in exosomes derived from MSCs with IL-1b promoted macrophage polarization to the M2 phenotype, reduced inflammation, and increased the survival of mice [66]. Using an IL-10 KO mouse model of systemic inflammation, endothelial progenitor cell exosomes improved endothelial cell proliferation and tube formation, and inhibited apoptosis. With IL-10 deficiency, impaired function was observed. Modulation of enriched miR-375 rescued IL-10KO-EPC-Exo dysfunction [67].

### 3.3. Neurological Disease

Only 13 articles included in this study were related to neurological diseases of miRNA in exosomes. Neurological diseases are associated with nervous system disorders. Here, we have discussed the roles or attenuation of miRNAs in exosomes in neurological diseases (Table 3).

In a mouse with middle cerebral artery occlusion used for cerebral infarction model, exosome-miR-542 derived from MSCs suppressed cerebral injury and inflammation by inhibiting TLR4 [68]. In a stress-induced depression mouse model, levels of BDNF, TrkB, and synaptotagmin 1 were decreased in the hippocampus, PFC, and serum exosomes. The miRNA profiling revealed that differentially expressed miRNAs were possibly involved in the pathogenesis of depression through the MAPK, Wnt, and mTOR pathways [69]. In an amyotrophic lateral sclerosis mouse model, miR-467f and miR-466q associated with MSC-derived s-EV reduced neuroinflammation. Furthermore, miR-467f and miR-466q reduce the activation of p38 MAPK signaling by inhibiting Map3k8 and Mk2 [70]. Using a repetitive traumatic brain injury mouse model, a study showed that miR-124 in exosomes improved neurologic outcomes and inhibited neuroinflammation by targeting PDE4B, thus suppressing mTOR signaling [71]. A study on a mouse model of endotoxemia induced by LPS that stimulated neuroinflammation revealed that the inflammatory cytokine mRNA, miR-155, and systemic inflammatory cytokine production increased. The serum-derived exosomes elevated inflammation-related miRNAs, such as miR-21, miR-125a, miR-146a, and miR-155. These miRNAs were engaged in modulating TLR signaling [72]. Using a chronic mild stress mouse model of depression, NK cell-derived exosomes carrying miR-207 alleviated symptoms such as depression and decreased pro-inflammatory cytokines, targeted TLR4, and hence an inhibited NF-κB signaling in astrocytes [73]. In a rat model of spinal cord injury, miR-544 in exosomes derived from BMSC attenuated histological deficits and neuronal loss, and inhibited inflammation induced by spinal cord injury [74]. Another study used a rat model of spinal cord injury and revealed that exosomes improved neuroprotective effects through the miR-219a-2/YY1 axis [75]. In a mouse model of spared nerve injury, miR-21 antagomir in the dorsal root ganglia reduced pro-inflammatory macrophage infiltration, and miR-21 deletion in sensory neurons reduced neuropathic hypersensitivity [76]. For an ischemic brain injury rat model established by middle cerebral artery occlusion, miR-181c in the cortical neuron released exosomes that inhibited neuroinflammation by suppressing CXCL1 [77]. In a mouse model with KA-induced epileptic seizures, circHivep2 exosomes prevented microglial cell activation and inflammatory factors through the miR-181a/SOCS2 mechanism [78]. In a mouse model of Alzheimer’s disease induced by LPS, miR-146a was enriched in EVs under inflammatory conditions, and EVs can induce inflammation and LPS tolerance [79]. Using the APP/PS1 mouse model of Alzheimer’s disease, miR-22 in exosomes from ADMSC enhanced neurological function, inhibited PC12 apoptosis, and decreased inflammatory factors by inhibiting proptosis [80].

### 3.4. Metabolic Syndrome

Only seven of the ninety-five articles explored were related to metabolic syndromes involving miRNAs of exosomes. Metabolic syndrome increases the risk of heart disease, diabetes, and other health problems. Here, we determined how miRNAs in exosomes play a role in metabolic syndrome (Table 4).

In a streptozotocin (STZ)-induced diabetic rat model, miR-21 exosomes from MSCs promoted ulceration repair, ischemic hindlimb blood perfusion, ischemic repair, and angiogenesis [81]. Using a non-diabetic NOD mouse model of type 1 diabetes, the increase in serum EV miR-21 preceded hyperglycemia and circulating EV miR-21 could be a biomarker of developing type 1 diabetes [82]. In a mouse model, miR-17 containing hucMSCs-derived exosomes alleviated oxidative injury by inhibiting STAT1 [83]. In a mouse model of dietary obesity induced by STC nutrition and a high-fat diet (HFD), miR-34a of adipocyte-secreted exosomal vesicles led to obesity-induced metabolic dysfunction and M2 macrophage proliferation by inhibiting KLF4 [84]. Using the plasma exosomes from a glycogen storage disease type 1a mouse model, differentially expressed miRNAs were correlated with various pathologic liver states and circulating miRNAs could be a biomarker of glycogen storage disease type 1a [85]. In the case of a type 2 diabetic mouse, miR-29 promoted inflammation and diabetes via TRAF3 [86]. In an obese mouse model established by feeding a HFD, miR-690 in exosomes from M2-polarized bone marrow-derived macrophages improved insulin sensitivity via NADK [87].

### 3.5. Vesicular Disease

Among the 95 articles, only 8 articles were associated with miRNAs exosomes in vesicular disease. Vesicular disease is a kind of blood vessel disorder, which can occur in the location of different types of artery and veins. We organized the relations between miRNA exosomes and versicular diseases (Table 5).

In a mouse model of atherosclerotic diabetes, EPC-derived exosomes and its miRNAs ameliorated diabetic atherosclerotic plaques, endothelial dysfunction, and inflammatory factors [88]. In an atherosclerosis mouse model established by feeding a HFD, IRES-Il-10 mRNA carried in exosomes can be activated by miR-155 and alleviate local inflammation [89]. miR-512 enriched by MSC-derived exosomes had a protective effect on EC cells against oxidized low-density lipoprotein via targeting KEAP1 [90]. In the same HFD-induced atherosclerosis mouse model, hUCMSC-derived exosomes carrying miR-100 decreased the atherosclerotic plaque area and inflammation via FZD5/WNT/β-catenin pathway [91]. In a mouse model of hypoxic pulmonary hypertension, MSC-derived exosomes inhibited STAT3 and increased the miR-17 microRNA superfamily [92]. In another HFD-induced atherosclerosis mouse model, MSCs-exosomes suppressed the atherosclerotic plaque area and macrophage infiltration via let-7/HMGA2/NF-kB pathway [93]. In an atherosclerosis mouse model established by feeding a HFD, miR-146a derived from oxidized low-density lipoprotein treated THP-1 cells exosomes enhanced the atherosclerotic plaque area and led to atherosclerosis deterioration via targeting SOD2 [94]. In the rat myocardial ischemia-reperfusion injury model, miR-98 in exosomes from hypoxic BMSCs promoted cardiac function and suppressed the inflammation response by targeting TLR4, and thus activating the PI3K/Akt signaling pathway [95].

### 3.6. Arthritis

Six of the ninety-five articles studied were related to miRNA exosomes involved in arthritis. Arthritis is a disorder that commonly affects joints. This usually makes it difficult for them to be active. We examined how exosomal miRNAs influence arthritis (Table 6).

In a mouse model of mBSA-induced arthritis, the injection of anti-miR-132 attenuated inflammatory arthritis [96]. In a mouse model of osteoarthritis, miR-206 in exosomes from BMSC promoted proliferation and osteoblast differentiation by inhibiting ELF3 [97]. In another rat model of arthritis, miR-223 in exosomes from BMSCs regulated inflammasome activation by inhibiting NLRP3 [98]. Using an osteoarthritis rat model established by surgery, researchers found that miR-361 from hBMSC-derived exosomes alleviated chondrocyte damage by inhibiting DDX20; thus, the NF-κB signaling pathway was inactivated [99]. In a collagen-induced arthritis (CIA) mouse model, miR-146a in MSC-derived exosomes increased FOXP3, TGFβ, IL-10, and miR-155 levels that increased RORγt, IL-17, and IL-6 levels. Such modulations altered Treg cell levels and possibly improved the recovery of appropriate T-cell responses in RA [100]. In a CIA rat model, miR-192 in exosomes developed from BMSCs reduced the inflammatory response by targeting RAC2 [101].

### 3.7. Cancer

Only five of the ninety-five articles we studied were associated with roles of miRNA exosomes in cancer. Cancer is a type of disease with abnormally increased growth of cells that spreads easily, commonly leading to low survival rates. We discussed the relationships between the different types of cancer and miRNAs in exosomes and determined the underlying mechanism (Table 7).

In a mouse model, tumor growth was decreased by miR-155, whereas miR-205 increased tumor growth. miR-155 and miR-205 play important roles in tumor growth of breast cancer cells in mice [102]. In a breast tumor mouse model, miR-183 in exosomes derived from tumor cells decreased tumor growth and promoted pro-inflammatory cytokines by targeting PPP2CA [103]. In a tumor initiated by xenografts of lung tumor cells of mice, the injection of miR-101 suppressed lung tumor growth, macrophage tumor infiltration, and inflammation and inhibited CDK8 and Ki-67 expression [104]. In an intracranial mouse glioma model induced by injections of GL261 glioma cells, uptake of glioma cell-released EVs by microglia and monocytes/macrophages in the brain increased miR-21, decreased c-Myc mRNA, and increased the proliferation of mouse microglia [105]. In an azoxymethane/DSS-induced colitis-associated colorectal cancer model, miR-146a transfected into hucMSC-derived exosomes alleviated cancer progression by inhibition of SUMO1 [106].

### 3.8. Other Inflammatory Diseases

Eleven articles were discussed that dealt with other inflammatory diseases. We examined the mechanisms of action of miRNA exosomes in diverse diseases (Table 8).

In a ligature-induced periodontitis and diet-induced obesity mouse model, an miR-25 inhibitor suppressed local inflammation, and exosomes of miR-25 in saliva contributed to the advancement of diabetes-associated periodontitis [107]. Using a rat model of skull defects, a study found that exosomes from adipose-derived stem cells improved bone healing and regulated M1/M2 macrophage polarization via the miR-451a/MIF axis [108]. In a femoral defect model established by surgery, miR-181b in exosomes promoted osteointegration and suppressed the inflammatory response via the PRKCD/AKT axis [109]. In an acute graft-versus-host disease mouse model, miR-223 in exosomes derived from MSCs inhibited inflammatory cytokines and attenuated disease progression by suppressing donor T-cell migration [110]. A study using a tendon defect model showed that miR-144 enriched in exosomes from tendon-derived stem cells improved the injured tendons. The performance of biomechanical testing was enhanced by targeting ARID1A [111]. In a cisplatin-induced hearing loss mouse model, miRNAs such as miR-125a, miR-125b, and miR-127 were highly abundant in UCMSC exosomes that improved hearing loss through reduced cochlear hair cell loss [112]. In a dimethylbenzanthracene-induced oral potentially malignant disorder (OPMDs) hamster model, miR-185 in extracellular vesicles derived from MSCs alleviated the inflammatory response and suppressed the progression of OPMDs by targeting AKT [113]. In a mouse model of intervertebral disc degeneration, established using the puncture method and H2O2 exposure, platelet-rich plasma exosomes enriched with miR-141 suppressed IVD degeneration by activating the KEAP1/NRF2 pathway [114]. In preterm birth induced by lipopolysaccharides (LPS), miR-146a levels were elevated. miR-146a and miR-548e from amniotic fluid-derived MSCs showed anti-inflammatory effects on human trophoblasts [115]. In a periodontitis rat model induced by LPS, miR-17 in periodontal ligament stem cells was suppressed by inflammation and alleviated its target VEGFA [116]. In an IL-1β-induced intervertebral disc degeneration model, miR-142 in exosomes derived from BMSCs alleviated NPC injury by targeting MLK3, thus inhibiting MAPK signaling [117].

## 4. Conclusions

Experimental studies of EVs and miRNAs are newly emerging and are in demand. We focused on diverse EV-associated miRNAs that play crucial roles in various inflammatory diseases. In this review, we have discussed the association of miRNAs in EVs with inflammatory diseases in rodent models. EVs have been recognized as the cargo of various molecules transported from origin cells to recipient cells mostly in all organisms. EVs also play complex and important roles in the pathophysiology of several diseases. Moreover, diverse EV-associated miRNAs play crucial roles in various inflammatory diseases. Given the previously suggested functions of EVs and increasing interest in clinical implications of EVs in various diseases, this study focused on miRNA in EVs in inflammatory diseases to further analyze their involvement in inflammatory responses. However, the studies included in this review are insufficient for comprehensive knowledge about the mode of action of extracellular miRNAs in inflammation. In addition, the included studies mostly reported changes in miRNA expression in different disease models, yet there was limited evidence of how the loss or gain of each miRNA function work in disease conditions. Further functional studies on each miRNA in inflammatory disease are needed to confirm the use of extracellular miRNAs as potential biomarkers or as a therapeutic method in which they can be safely and efficiently delivered to the target region. Nevertheless, our understanding suggests an opportunity for further study of extracellular miRNAs as biomarkers and the early diagnosis of inflammatory diseases and disorders. Moreover, the use of EVs might further offer the possibility of gene therapeutic approaches for inflammation.

## Figures and Tables

**Table 1:** Extracellular miRNAs in organ injury.

	Author	Disease	Subject	EV Type	Targeted miRNAs
Liver	Bala et al. (2012) [23]	Alcoholic liver disease	Mouse	Exo	miR-122, miR-155,
	Chen et al. (2018) [24]	Autoimmune hepatitis	Mouse	Exo	miR-223
	Chen et al. (2018) [25]	Liver fibrosis	Mouse	EV	miR-34c, miR-151, miR-483, miR-532, miR-687
	Liu et al. (2018) [26]	Acute liver failure	Mouse	Exo	miR-17
	Liu et al. (2020) [27]	Nonalcoholic fatty liver disease	Rat	Exo	miR-192
	Lu et al. (2019) [28]	Autoimmune hepatitis	Mouse	Exo	miR-223
	Shao et al. (2020) [29]	Acute liver injury	Mouse	Exo	miR-455
Lung	Jiang et al. (2021) [30]	Acute lung injury	Mouse	Exo	miR-125b
	Liu et al. (2021) [31]	Acute lung injury	Mouse	Exo	miR-384
	Shen et al. (2022) [32]	Septic lung injury	Mouse	Exo	miR-490
	Tian et al. (2021) [33]	Septic lung injury	Mouse	Exo	miR-16
	Wei et al. (2020) [34]	Acute lung injury	Mouse	Exo	miR-377
	Zhang et al. (2019) [35]	Lung inflammation	Mouse	MV	miR-223, miR-142
	Zheng et al. (2021) [36]	Acute lung injury	Rat	Exo	miR-22
Heart	Chen et al. (2017) [37]	Myocardial infarction	Rat	Exo	miR-133
	Kwon et al. (2021) [38]	Myocardial infarction	Rat	EV	miR-7004, mi7-7b
	Luo et al. (2017) [39]	Acute myocardial infarction	Rat	Exo	miR-126
	Milano et al. (2020) [40]	Cardiotoxicity	Rat	Exo	miR-146a
	Pan et al. (2019) [41]	Myocardial infarction	Rat	Exo	miR-146a
	Peng et al. (2020) [42]	Myocardial infarction	Mouse	Exo	miR-25
	Sun et al. (2022) [43]	Sepsis induced myocardial infarction	Mouse	Exo	miR-24
	Wang et al. (2015) [44]	Sepsis induced myocardial dysfunction	Mouse	Exo	miR-223
	Wang et al. (2022) [45]	Myocardial infraction	Mouse	Exo	miR-129
	Yu et al. (2021) [46]	Cardiac hypertrophy	Rat	Exo	miR-155
Bowel	Cai et al. (2021) [47]	Colitis	Mouse	Exo	miR-378a
	Deng et al. (2021) [48]	Ulcerative colitis	Mouse	Exo	miR-590
	Lu et al. (2021) [49]	Ulcerative colitis	Mouse	Exo	miR-21a
	Sun et al. (2020) [50]	Inflammation-injured IEC	Rat	Exo	miR-200b
Kidney	Li et al. (2019) [51]	Tubulointerstitial inflammation	Mouse	Exo	miR-23a
	Li et al. (2020) [52]	Ischemia/reperfusion injury	Rat	Exo	miR-146a
	Li et al. (2020) [53]	Diabetic kidney diseases	Mouse	Exo	miR-26a
	Pan et al. (2019) [54]	Sepsis, Acute kidney injury	Mouse	Exo	miR-21
Other organs	Jimenez-Alesanco et al. (2019) [55]	Acute pancreatitis	Rat	Exo	miR-155
	Liang et al. (2019) [56]	Urethral stricture	Rat	Exo	miR-146a
	Liu et al. (2021) [57]	Intrauterine adhesion	Mouse	Exo	miR-223

Exo: exosome; EV: extracellular vesicle; MV: micro vesicle.

**Table 2:** Extracellular miRNAs in immune dysfunction.

Author	Disease	Subject	EV Type	Targeted miRNAs
Alexander et al. (2017) [58]	Chronic inflammation	Mouse	Exo	miR-155
Appiah et al. (2021) [59]	Sepsis	Mouse	EV	miR-146a, miR-9, and miR-155
Balusu et al. (2016) [60]	Systemic inflammatory diseases	Mouse	EV	miR-1a, miR-9, miR-146a, miR-155
Fernández-Messina et al. (2020) [61]	Immune diseases	Mouse	EV	miR-20a, miR-25, miR-155
Gao et al. (2021) [62]	Sepsis	Rat	Exo	miR-1
Li et al. (2021) [63]	Asthma	Mouse	Exo	miR-370
Okoye et al. (2014) [64]	Systemic disease	Mouse	Exo	let-7d
Shan et al. (2022) [65]	Asthma	Mouse	Exo	miR-188
Song et al. (2017) [66]	Sepsis	Mouse	Exo	miR-146a
Yue et al. (2017) [67]	Systemic inflammation	Mouse	Exo	miR-375

Exo: exosome; EV: extracellular vesicle.

**Table 3:** Extracellular miRNAs in neurological disease.

Author	Disease	Subject	EV Type	Targeted miRNAs
Cai et al. (2021) [68]	Ischemic stroke	Mouse	Exo	miR-542
Fang et al. (2020) [69]	Depression	Rat	Exo	miR-455, miR-126a, miR-122, miR-1b
Giunti et al. (2021) [70]	Neuroinflammation	Mouse	Exo	miR-467f, miR-466q
Huang et al. (2018) [71]	Traumatic brain injury—neuronal inflammation	Mouse	Exo	miR-124
Li et al. (2018) [72]	Neuroinflammation	Mouse	Exo	miR-21, miR-125a, miR-146a, miR-155
Li et al. (2020) [73]	Depression	Mouse	Exo	miR-207
Li et al. (2020) [74]	Spinal cord injury	Rat	Exo	miR-544
Ma et al. (2019) [75]	Spinal cord injury	Rat	Exo	miR-219a-2
Simeoli et al. (2017) [76]	Neuropathic pain	Mouse	Exo	miR-21-5p, miR-21
Song et al. (2019) [77]	Ischemic brain injury	Rat	Exo	miR-181c
Xiaoying et al. (2020) [78]	Epilepsy	Mouse	Exo	miR-181a
Yang et al. (2021) [79]	Alzheimer’s disease	Mouse	Exo	miR-146a
Zhai et al. (2021) [80]	Alzheimer’s disease	Mouse	Exo	miR-22

Exo: exosome; EV: extracellular vesicle.

**Table 4:** Extracellular miRNAs in metabolic syndrome.

Author	Disease	Subject	EV Type	miRNAs
Huang et al. (2021) [81]	Ischemic disease—diabetic foot	Rat	Exo	miR-21
Lakhter et al. (2018) [82]	Type 1 diabetes	Mouse	EV	miR-21
Li et al. (2021) [83]	Diabetic retinopathy	Mouse	Exo	miR-17
Pan et al. (2019) [84]	Obesity-induced metabolic inflammation	Mouse	Exo	miR-34a
Resaz et al. (2020) [85]	Glycogen storage disease type 1a	Mouse	Exo	let-7d, miR-142, let-7i, miR-145a, miR-150, miR-15b, miR-192, miR-21a, miR-29a, miR-342, miR-345, miR-409, miR-486a, miR-744
Sun et al. (2021) [86]	Type 2 diabetes mellitus	Mouse	Exo	miR-29
Ying et al. (2021) [87]	Obesity	Mouse	Exo	miR-690

Exo: exosome; EV: extracellular vesicle.

**Table 5:** Extracellular miRNAs in vesicular disease.

Author	Disease	Subject	EV Type	miRNAs
Bai et al. (2020) [88]	Atherosclerosis	Mouse	Exo	miR-21a, miR-222, miR-221, miR-155, miR-29a, miR-199a, miR-146a
Bu et al. (2021) [89]	Atherosclerosis	Mouse	Exo	miR-155
Chen et al. (2021) [90]	Atherosclerosis	Mouse	Exo	miR-512
Gao et al. (2021) [91]	Atherosclerosis	Mouse	Exo	miR-100
Lee et al. (2012) [92]	Pulmonary hypertension	Mouse	Exo	miR-204
Li et al. (2019) [93]	Atherosclerosis	Mouse	Exo	let-7
Zhang et al. (2019) [94]	Atherosclerosis	Rat	Exo	miR-146a
Zhang et al. (2021) [95]	Coronary artery disease	Rat	Exo	miR-98

Exo: exosome; EV: extracellular vesicle.

**Table 6:** Extracellular miRNAs in arthritis.

Author	Disease	Subject	EV Type	miRNAs
Donate et al. (2021) [96]	Rheumatoid arthritis	Mouse	EV	miR-132
Huang et al. (2021) [97]	Osteoarthritis	Mouse	Exo	miR-206
Huang et al. (2022) [98]	Rheumatoid arthritis	Rat	Exo	miR-223
Tao et al. (2021) [99]	Osteoarthritis	Rat	Exo	miR-361
Tavasolian et al. (2020) [100]	Rheumatoid arthritis	Mouse	Exo	miR-146a, miR-155
Zheng et al. (2020) [101]	Rheumatoid arthritis	Rat	Exo	miR-192

Exo: exosome; EV: extracellular vesicle.

**Table 7:** Extracellular miRNAs in cancer.

Author	Disease	Subject	EV Type	miRNAs
Gorczynski et al. (2017) [102]	Breast cancer	Mouse	Exo	miR-155, miR-205
Guo et al. (2020) [103]	Breast cancer	Mouse	Exo	miR-183
Li et al. (2021) [104]	Lung cancer	Mouse	Exo	miR-101
Van der Vos et al. (2016) [105]	Glioblastoma	Mouse	EV	miR-21
Wang et al. (2022) [106]	Colorectal cancer	Mouse	Exo	miR-146a

Exo: exosome; EV: extracellular vesicle.

**Table 8:** Extracellular miRNAs in other inflammatory disease model.

Author	Disease	Subject	EV Type	miRNAs
Byun et al. (2022) [107]	Periodontitis	Mouse	Exo	miR-25
Li et al. (2022) [108]	Traumatic bone defects	Rat	Exo	miR-451a
Liu et al. (2021) [109]	Aseptic loosening and poor osteointegration	Mouse/rat	Exo	miR-181b
Liu et al. (2021) [110]	Acute graft-versus-host disease	Mouse	Exo	miR-223
Song et al. (2022) [111]	Tendon pathologies	Rat	Exo	miR-144
Tsai et al. (2021) [112]	Ototoxicity-induced hearing loss	Mouse	Exo	miR-125a, miR-125b, miR-127
Wang et al. (2019) [113]	OPMD	Hamster	EV	miR-185
Xu et al. (2021) [114]	Intervertebral disc degeneration	Mouse	Exo	miR-141
Yang et al. (2019) [115]	Placental oxidative stress, preterm birth	Mouse	Exo	miR-146a, miR-548e
Zhang et al. (2020) [116]	Periodontitis	Rat	Exo	miR-17
Zhu et al. (2020) [117]	Intervertebral disc degeneration	Mouse	Exo	miR-142

Exo: exosome; EV: extracellular vesicle.

## Data Availability

Not applicable.
